# Editorial: The cardiovascular continuum between hypertension, diabetes and cardiovascular disease

**DOI:** 10.3389/fcvm.2026.1813448

**Published:** 2026-03-11

**Authors:** Giuseppe Armentaro, Mario Daidone

**Affiliations:** 1 Geriatrics Division, Renato Dulbecco" University Hospital of Catanzaro, Catanzaro, Italy; 2 Internal Medicine and Stroke Care Ward, Department of Health Promotion, Mother and Child Care, Internal Medicine and Medical Specialties, University of Palermo, Palermo, Italy

**Keywords:** arterial hypertension (AH), cardiovascular disease, heart failure, insulin resistance, type 2 diabetes mellitus

Hypertension, type 2 diabetes mellitus (T2DM) and cardiovascular disease (CVD) share pathophysiological pathways, mutually enhance each other and together define the cardiovascular continuum. This research topic brings together eleven original and review articles that collectively explore this continuum from a global epidemiological perspective to molecular mechanisms, while addressing the persistent clinical challenges of therapeutic inertia, residual risk and cardiometabolic multimorbidity.

## A growing global burden

The epidemiological scale of the cardiometabolic continuum is established by two large-scale analyses anchored to the Global Burden of Disease 2021 dataset. Peng et al. quantify the fraction of hypertensive heart disease (HHD) mortality attributable to metabolic risk factors across 204 countries between 1990 and 2021, documenting a rise from 13.4% to 16.9% over three decades and identifying elevated systolic blood pressure and high BMI as the dominant drivers, with an excess burden concentrated in older women. Mapping the arterial periphery, Li et al. apply time-series modelling to GBD 2021 incidence data for lower-extremity peripheral arterial disease, projecting continued growth especially in lower-middle-income regions of South Asia and sub-Saharan Africa — precisely where diabetes and hypertension prevalence is accelerating. Together, these studies define the epidemiological urgency that frames the entire Research Topic.

## Pathophysiological mechanisms: from insulin resistance to organ damage

A coherent mechanistic thread links three of the contributions. Zhong et al. demonstrate, in non-diabetic STEMI patients undergoing PCI, that subclinical insulin resistance — regardless the presence of T2DM — independently predicts impaired coronary microcirculatory function and increased major adverse cardiovascular events (MACE) during follow-up. This finding confirms the hypothesis that insulin resistance is a cardiovascular risk factor, independent of T2DM. Chen et al. reinforce this perspective with a systematic review of T2DM-mediated heart failure, tracing the cascade from chronic hyperglycaemia and advanced glycation end-product accumulation to diabetic cardiomyopathy and heart failure with preserved ejection fraction (HFpEF) — a phenotype that frequently eludes conventional diagnostic algorithms. Complementing these mechanistic insights, Song et al. analyse a randomised trial using hierarchical composite endpoints to evaluate intensive glycaemic control and kidney disease risk, illustrating the complex trade-off between microvascular protection and hypoglycaemia hazard that clinicians must navigate when individualising glucose targets.

## Multimorbidity and the accumulation of sub-threshold risk

The non-linear amplification of risk when multiple cardiometabolic conditions coexist is examined from complementary angles. Wu et al. present a systematic review and meta-analysis confirming that the relationship between BMI and cardiometabolic multimorbidity begins in the overweight range (25–30 kg/m^2^), well before obesity thresholds are reached, with each incremental BMI unit carrying additional risk. Chehal et al. extend this observation to a socially vulnerable population, documenting rising multimorbidity prevalence among non-elderly enrolled between 2018 and 2022, highlighting equity dimensions that aggregate statistics often obscure. At the other end of the age spectrum, Fangman et al. report that Type D personality — characterised by negative affectivity and social inhibition — is associated with early markers of vascular dysfunction in adolescents, underscoring that the cardiovascular continuum begins to take shape long before clinical risk factors manifest. The conceptual apex of this section is the perspective by López-Gil et al., who introduce the *Gulliver syndrome*: a framework to capture the clinically significant yet systematically overlooked risk conferred by the simultaneous presence of at least four borderline risk factors — each sub-threshold by conventional guidelines, yet collectively imposing a cardiovascular burden analogous to established disease. The metaphor, drawn from Swift's protagonist restrained by countless small threads, elegantly frames the problem of therapeutic inertia in patients who fall below every single action threshold. Unlike the metabolic syndrome, the Gulliver syndrome does not require central obesity as a defining feature, making it applicable to a wider range of phenotypes.

## Risk stratification, endpoints, and the evidence base

Two methodologically oriented contributions address the tools we use to measure and act upon cardiovascular risk in T2DM. Guo and Wu compare cardiovascular risk stratification protocols in a real-world diabetic cohort, finding that protocol choice substantially alters patient classification and that simultaneous attainment of blood pressure, HbA1c, and LDL-cholesterol targets remains the exception rather than the rule — a stark reminder of the implementation gap between guidelines and practice. Van Bruggen and Luijendijk raise an important methodological concern: the use of type 2 myocardial infarction (demand-supply mismatch rather than plaque rupture) as an efficacy endpoint in cardiovascular trials may conflate mechanistically distinct events and distort treatment effect estimates, with downstream consequences for guideline recommendations.

## Conclusion

Taken as a whole, the eleven articles assembled in this Research Topic articulate a compelling and consistent message: hypertension, T2DM, and CVD are facets of a single, temporally extended pathological process. Acting on this continuum requires moving beyond single-disease, single-threshold logic — integrating psychosocial determinants, early-life exposures, and cumulative sub-threshold risk into a genuinely unified clinical approach. The Editors hope that these contributions will stimulate both further research and the translation of this integrative framework into everyday cardiovascular prevention practice.

